# Safety and Toxicology Study of Hu7691, a Novel AKT Inhibitor, following Oral Administration in Rats

**DOI:** 10.3390/toxics11110880

**Published:** 2023-10-26

**Authors:** Renhua Gai, Chao Chen, Wei Zhang, Jian Ma, Xiaomeng Wang, Xiaoqing Chi, Guangxing Li

**Affiliations:** 1 College of Veterinary Medicine, Heilongjiang Key Laboratory for Animal and Comparative Medicine, Northeast Agricultural University, Harbin 150030, China; gairenhua@zju.edu.cn; 2 Center for Drug Safety Evaluation and Research, College of Pharmaceutical Sciences, Zhejiang University, Hangzhou 310058, China; snowman2034@zju.edu.cn (C.C.); 0917445@zju.edu.cn (W.Z.); majianfly@zju.edu.cn (J.M.); wangxiaomeng@zju.edu.cn (X.W.); tiffany1127@zju.edu.cn (X.C.)

**Keywords:** Hu7691, 14-day exposure, oral toxicity study, SD rats, formulation, histopathology

## Abstract

Hu7691 represents a novel Pan-Akt kinase inhibitor, demonstrating excellent selectivity towards non-AGC kinase families and pronounced inhibitory effects on the proliferation of multiple tumor cell lines. However, there is currently a notable absence of in vivo toxicological research evidence concerning Hu7691. This study represents the first investigation into the 14-day repeated-dose toxicity of Hu7691 in male and female Sprague Dawley (SD) rats. Male rats were administered daily doses of 12.5, 50, 100, and 150 mg/kg/day, while female rats received doses of 12.5, 25, 50, and 75 mg/kg/day for 14 consecutive days. Hematological assessments, organ weights, and histopathological examinations revealed corresponding alterations, suggesting potential target organs for toxicity including the spleen, thymus, and gastrointestinal tract. It is worth noting that the test substance may also impact the liver, kidneys, heart, and ovaries. The No Observed Effect Level (NOAEL) was determined to be no greater than 12.5 mg/kg/day. Based on the observed gender-related toxicity differences in preliminary trials, it is recommended that the high dose reference dose for male animals in formal experiments should not be less than 100 mg/kg/day, while for female animals, it should be less than 50 mg/kg/day.

## 1. Introduction

Cancer stands as one of the most formidable challenges in human medicine. Presently, the global landscape of oncological therapeutics is rapidly advancing towards the era of precision medicine, with the key components being genetic sequencing, tumor profiling, and personalized treatment. Currently, the most mature domain within precision medicine primarily revolves around targeted therapies, exemplifying the treatment of cancer with drugs tailored to specific molecular targets [1]. The phosphatidylinositol 3-kinase/protein kinase B/mammalian target of the rapamycin (PI3K/Akt/mTOR) pathway represents a critical signaling axis regulating various biological events in cancer, including proliferation, apoptosis, and angiogenesis. Aberrant activation of this pathway plays a pivotal role in the malignant progression of tumors. Consequently, it stands as a prominent focus in current research and development efforts for novel anti-cancer therapies [2,3,4,5]. Clinical data indicate that in over 30% of malignant tumors, the PI3K/Akt/mTOR signaling pathway is aberrantly activated. Substantial laboratory evidence further supports the notion that inhibiting this signaling pathway can effectively suppress tumor growth [6,7,8,9,10,11,12,13,14].

Akt is a serine/threonine protein kinase, and the Akt family primarily consists of three subtypes: Akt1, Akt2, and Akt3. These subtypes are closely related and play a pivotal role in regulating cell growth, proliferation, survival, and metabolism. They are central proteins within the PI3K/Akt/mTOR signaling pathway [15]. Following PI3K activation, the interaction between PIP3 and the PH domain of Akt occurs. This interaction leads to the translocation of Akt from the cytoplasm to the cell membrane, accompanied by a conformational change in Akt that exposes its serine and threonine residues. Subsequently, PIP3 binds to the PH domain of Akt, leading to the phosphorylation of serine residues by PDK1 and threonine residues by PDK2. Only when both serine and threonine residues are phosphorylated does Akt become activated. Activated Akt then shuttles between the cell membrane, cytoplasm, and nucleus. It continues to modulate downstream signaling molecules through phosphorylation, including mTOR, Bad, cyclin D1, and various other cellular proteins, promoting cell growth and inhibiting apoptosis [16].

**Figure 1:**
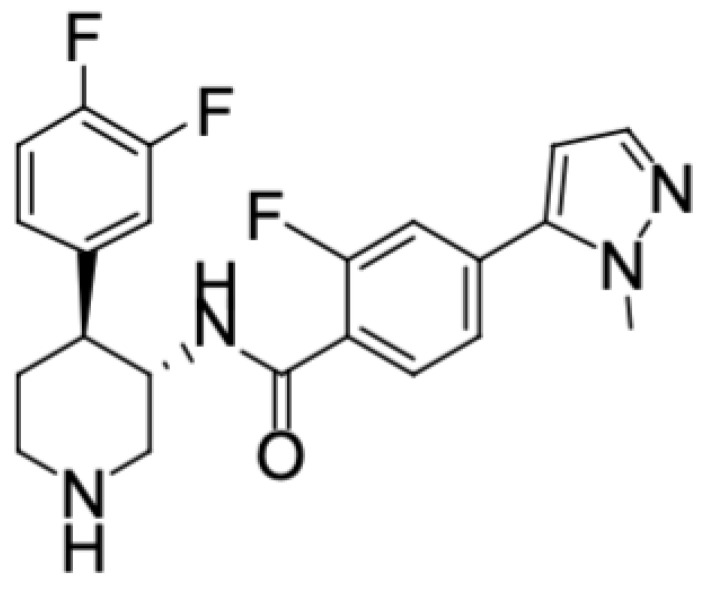
Structure of Hu7691.

Hu7691 is a novel Pan-Akt kinase inhibitor (Figure 1), showing significant suppression in neuroblastoma, gastric cancer, osteosarcoma, and renal cancer xenograft models. Hu7691 holds promise as a prospective candidate for clinical drug development [17]. However, currently, there is a lack of toxicological information. This study explores the possible toxic effects of Hu7691 for the first time, and evaluates and determines the toxicity response of the test substance in SD rats exposed to different doses of Hu7691 for 14 days, providing a certain toxicological reference for clinical research and trials of investigational new drugs (INDs).

## 2. Materials and Methods

### 2.1. Test Article and Chemicals

Hu7691 (97.98% purity) was obtained from Zhejiang University a College of Pharmaceutical Sciences (Hangzhou, China). Dose formulations were prepared with cosolvents. All dosing solutions were currently prepared before administration. After preparation, Hu7691 remained stable after stirring at room temperature for 6 h. After preparation, storage at 2–8 °C for 92 h, being left at room temperature for 30 min, and stirred for 6 h, Hu7691 remains stable. The measured concentrations of Hu7691 administration formulations at various concentrations are within 85%–115% of the indicated concentration, with an RSD not exceeding 10%. Cosolvents, Methyl cellulose M450 were purchased from Sinopharm Chemical Reagent Co., Ltd. (Shanghai; China). 

### 2.2. Experimental Animals

SD rats (six weeks old) were purchased from Hangzhou Vital River Laboratory Animal Technologies Co., Ltd. (Hangzhou, China). There were no significant abnormalities in animals during the acclimatization period. It was indicated that the batch of laboratory animals could be used in this study. Feed and water were supplied ad libitum, and alternated light and dark every 12 h. The room temperature and humidity of animal rooms were set at 22 ± 3 °C and 50 ± 10%, respectively. The animal use application (AUP) for this study has been approved by the Institutional Animal Care and Use Committee (IACUC; Hangzhou, China), and the IACUC No. was IACUC-18-286. 

### 2.3. Experimental Design

According to the previous study, the maximum tolerated dose of Hu-7691 was 50 mg/kg/day in male and 25 mg/kg/day in female animals, respectively. The dose of Hu-7691 used in this study where 200 mg/kg/day or 600 mg/kg/day in male and 100 mg/kg/day or 150 mg/kg/day in female animals was found applicable to induce perianal filth, weight loss and food consumption reduction. The previous study showed that Hu-7691 could induce gastrointestinal toxicity, thymic toxicity and cardiotoxicity.

Thirty rats were randomly divided into 5 groups, each consisting of 6 rats (3/sex/group). The male rats in groups 2 to 5 orally received Hu-7691 12.5 mg/kg/day, 50 mg/kg/day, 100 mg/kg/day and 150 mg/kg/day for 14 days, respectively. The female rats in groups 2 to 5 orally received Hu-7691 12.5 mg/kg/day, 25 mg/kg/day, 50 mg/kg/day and 75 mg/kg/day for 14 days, respectively. Group 5 was given 0.5% MC and set as control. Body weights were measured on D1, D4, D8, D11 and D14 before and post administration. Average food consumption was calculated weekly. Blood samples were collected on D14 before necropsy for analysis of hematology and biochemistry parameters. Organs were collected after necropsy for organ weight ratio calculation and histopathology observation.

### 2.4. Clinical Observation 

The animals in each group had their clinical symptoms observed, including appearance, skin, behavior, gland secretion, respiration, eyes, ears, nose, anus and fecal properties, and limbs before administration once a day. 

### 2.5. Body Weights and Food Consumption

The animals were observed and recorded once a day, including morbidity and mortality. The body weights were examined before administration and on D1, D4, D8, D11, D14 post Hu7691 administration. Body weights were expressed as Mean ± SD according to sex of the animals. The total food consumption of each cage was examined and the average food consumption of each animal was calculated weekly.

### 2.6. Hematology and Biochemistry Analysis

Blood samples were collected from abdominal aorta via vein with a vacuum blood collection needle and put into the vacuum negative pressure blood collection vessel with anticoagulant EDTA-K_2_ (hematology examination), coagulant (biochemistry examination) or anticoagulant sodium citrate (coagulation examination) on D14. Before blood sample collection, all animals were fasted overnight. Then, hematology parameters were examined by an automated hematology analyzer Sysmex XT-2000i (Sysmex Corporation, Kobe, Japan) as follows: white blood cell count (WBC), red blood cell count (RBC), neutrophils (NEUT), lymphocytes (LYMPH), monocytes (MONO), eosinophils (EO), hemoglobin (HGB), hematocrit (HCT), mean corpuscular volume (MCV), mean corpuscular hematoglobin (MCH), mean corpuscular hematoglobin concentration (MCHC), blood platelet count (PLT), and reticulocyte count (RET#). 

Biochemistry parameters were examined on an automatic chemistry analyzer Cobas c311 (Roche Diagnostics, Basel, Switzerland) as follows: total protein (TP), albumin (ALB), alanine aminotransferase (ALT), aspartate aminotransferase (AST), total bilirubin (TB), alkaline phosphatase (ALP), blood urea nitrogen (BUN), glucose (GLU), triglyceride (TG), total cholesterol (TC), creatine kinase (CK), sodium (Na^+^), potassium (K^+^) and chloride (Cl^−^) ions.

Coagulation was examined on an automatic blood coagulation analyzer CA-1500 (Sysmex Diagnostics, Kobe, Japan) as follows: activated partial thromboplastin time (APTT), and thrombin time (TT).

### 2.7. Necropsy, Organ Weight and Histopathology

Before necropsy, animals in each group were fasted overnight and then profoundly anesthetized using pentobarbital sodium via intramuscular injection, and then euthanasia was performed by exsanguination after anesthesia, and was subsequently followed by a pathology examination for the necropsy. Tissues and organs were collected and weighed as follows: the liver, heart, kidneys, spleen, lungs, brain, cerebellum, thymus, adrenals, testicle, epididymis, ovaries and womb. The relative organ weights were calculated. The collected tissues were preserved in neutral buffered formalin solution. The tissues in the control and highest dose group, the abnormal tissues or organs in gross anatomy and the preserved tissues or organs in morbidity and mortality animals were dehydrated, embedded in paraffin, sliced and stained in hematoxylin and eosin for histopathological examination.

### 2.8. Statistical Analysis 

All collected data were organized by each dose group by sex. Data were expressed as Mean ± SD for the quantitative results. One-way analysis of variance (ANOVA; IBM, Amenk, NY, USA) was performed to assess differences in these continuous variables, followed by the Kruskal–Wallis test when it was non-parametric. The results listed the statistically significant differences between each dose group and control group for a *p*-value below 0.05. All statistical analyses were conducted using SPSS v26.0 (SPSS Inc., Chicago, IL, USA; IBM, Amenk, NY, USA).

## 3. Results

### 3.1. Clinical Observations

One animal (5M03) in the 150 mg/kg/day dose group died on Day 8(D8), one animal (5M01) in the 150 mg/kg/day dose group died on D9, one animal (4F01) in the 50 mg/kg/day dose group died, one animal (5F01) in the 75 mg/kg/day dose group died. On D5~D8, red staining around the nose, bristling hair, emaciation, and arched back were observed in the dead animals. Then, large gastrointestinal volumes, dilatation of the jejunum and duodenum, and fluid filling in the stomach, jejunum, and duodenum were subsequently observed.

Red-stained nose, arched back and emaciation were observed in some female animals at the dose group (Hu-7691 ≥ 25 mg/kg/day). 

The female animals at the dosage of ≥25 mg/kg/day showed red-stained nose, arched back and emaciation. The animals at the dosage of more than 50 mg/kg/day showed the symptom of vertical hair and matte hair, and the animals at the dosage of 75 mg/kg/day also showed the symptom of anal filth. At the dose of ≥100 mg/kg/day, the results showed the symptoms of vertical hair, dull hair, red nose, arched back, and emaciation of male animals. No other significant clinical changes were observed.

The clinical symptoms are shown in Table 1.

### 3.2. Body Weight

**Figure 2:**
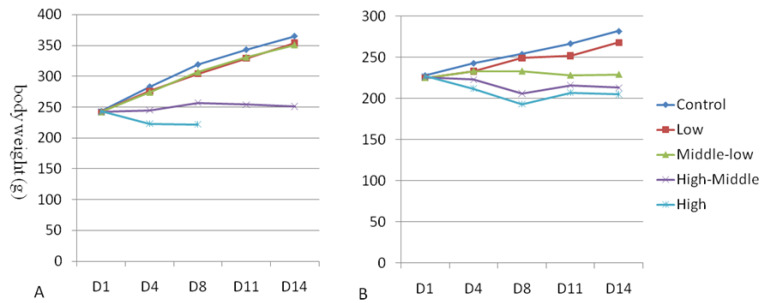
Mean body weights (g) of male (**A**) and female (**B**) rats orally exposed to Hu7691 for 14 days.

The mean body weight of male and female rats was calculated and is shown in Figure 2A,B. Compared with the vehicle control group, the dosage of ≥50 mg/kg/day in female animals was significantly decreased on D4~D14, and the dosage of 25 mg/kg/day in female animals was significantly decreased on D11~D14 (*p* < 0.05~0.01). The number of male animals at the dose of 150 mg/kg/day was significantly decreased on D4~D8 (the number of the animals at the dose 150 mg/kg/day group was one after D8, which did not meet the statistical criteria), and the number of the animals in the 100 mg/kg/day dose group significantly decreased on D4~D14 (*p* < 0.05~0.001) (Figure 2A,B).

### 3.3. Hematology

Hematology parameters were evaluated after Hu7691 administration in rats and provided in Table 2. Compared with the vehicle control group, the WBC in female animals at the dose ≥25 mg/kg/day significantly increased (*p* < 0.05). The levels of NEUT in male animals at the dose ≥ 50 mg/kg/day and in female animals at the dose ≥ 50 mg/kg/day significantly increased and the levels of LYMPH in male animals at the dose ≥ 50 mg/kg/day and in female animals at the dose ≥ 50 mg/kg/day significantly decreased (*p* < 0.05~0.001). There were significant Hu-7691 related changes on hematology during the administration period.

Additionally, the changes in Hb, MCV, MCH, MCHC and PLT levels were sporadic, not related to Hu7691 concentration, and within the normal range for the strain, which was not considered to be of biological relevance [18]. 

### 3.4. Serum Chemistry

Clinical blood biochemistry was examined after Hu7691 administration and summarized in Table 3. Most of these parameters did not alter after repeated administration of Hu7691. The BUN level was significantly decreased in all female rats exposed to Hu7691 (*p* < 0.05, *p* < 0.01, and *p* < 0.01, respectively), and the GLU level showed a significant decrease in all male rats exposed to Hu7691 compared with the control group (*p* < 0.01). Moreover, a significant increase in TB, Na^+^ ions, Cl^−^ ions levels occurred in all male rats receiving Hu7691 administration (*p* < 0.01, *p* < 0.01, and *p* < 0.05, respectively). Similarly, an obvious increase in TB, and Na^+^ ions levels was observed in female rats receiving 1000 mg/kg/day dose (*p* < 0.05), in comparison to the control group. 

### 3.5. Blood Coagulation Detection Index

Clinical blood biochemistry was examined after Hu7691 exposure and the data are summarized in Table 4. The Blood Coagulation Detection Index did not alter after repeated exposure to Hu7691.

### 3.6. Necropsy, Organ Weights and Histopathology

The gross anatomy of 3/3 male animals in the Hu7691 100 mg/kg/day dose group showed that the spleen and thymus were slightly smaller; bleeding points were observed in the glandular stomach, and duodenum and jejunum were dilated. The gross anatomy of the spleen and thymus in 2/3 of the 75 mg/kg/day dose group female animals is relatively small, while in 1/3 of the 50 mg/kg/day dose group female animals, the gross anatomy of the spleen and thymus is relatively small, and in 1/3 of the 25 mg/kg/day dose group female animals, the gross anatomy of the spleen and thymus is relatively small.

Compared with the solvent control group, the absolute weight, relative body weight coefficient, and relative brain weight coefficient of the spleen and thymus of male animals in the Hu7691 ≥ 50 mg/kg/day group were significantly reduced (*p* < 0.05–0.001), while the absolute weight and relative brain weight coefficient of the spleen and thymus of female animals in the 12.5 mg/kg/day and ≥50 mg/kg/day groups were significantly reduced (*p* < 0.05–0.001).

Compared with the solvent control group, the absolute weight and relative brain weight coefficients of the liver and kidneys of male animals in the Hu7691 100 mg/kg/day group decreased significantly (*p* < 0.01–0.001). The absolute weight and relative brain weight ratio of the heart in male animals at the dose of 100 mg/kg/day and female animals at a dose of 75 mg/kg/day decreased, while the absolute weight and relative brain weight coefficients of the ovaries in female animals at a dose of ≥ 25 mg/kg/day decreased, but no dose correlation was observed, The changes in these indicators cannot be ruled out to be related to the administration of the test substance.

The absolute organ weight, organ body weight coefficient, and organ brain weight coefficient data are shown in Table 5, Table 6 and Table 7.

**Figure 3:**
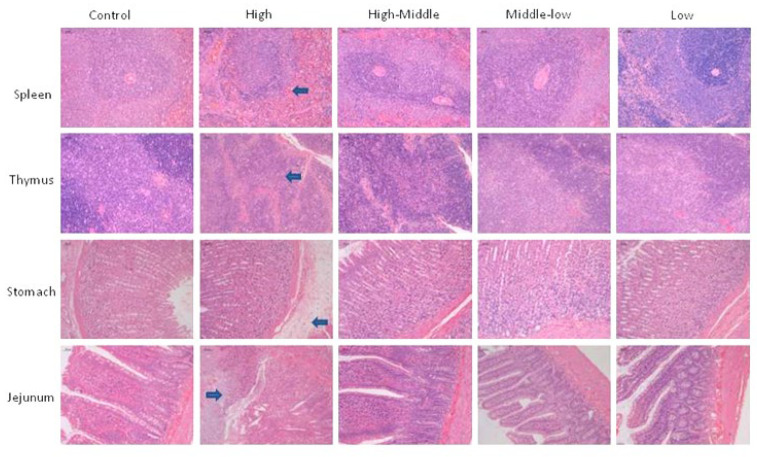
H&E stained sections of spleen, thymus, stomach and jejunum from SD rats treated with control and Hu7691 for 14 days (bars = 50 μm). The site of lesion is indicated by a blue arrow.

The spleen and thymus of male animals in HU7691 ≥ 100 mg kg group and female animals in HU7691 ≥ 50 mg kg group showed decreased lymphocyte cellularity. Gastric edema and jejunal ulcer were found in the HU7691 150 mg/kg/day group. No significant damage was observed in other animals (Figure 3).

## 4. Discussion

Akt serves as the central protein in the PI3K/Akt/mTOR signaling pathway. Hu7691, as a novel Akt kinase inhibitor, has shown excellent performance in our center’s research on anti-tumor efficacy. In our in vitro studies of its inhibitory effects on tumor cell proliferation, we have observed significant inhibitory effects on 21 different human tumor cell lines originating from various tissues, including glioblastoma, lung cancer, gastric cancer, and osteosarcoma (previous research conducted at our center, pending publication). Notably, these effects surpassed those of the positive reference. Moreover, in a 20-day oral administration study of Hu7691, it exhibited dose-dependent inhibition of tumor growth in nude mice with human gastric cancer HGC27, human osteosarcoma KHOS, and human renal cancer 786-O xenografts. Simultaneously, it showed a noticeable reduction in skin toxicity [19].

In this study, Hu7691 led to observable symptoms in rats, including piloerection, dull fur, redness around the nose, and kyphosis. Additionally, it caused a significant decrease in the body weight of the treated rats. Blood biochemical analysis results indicated that Hu7691 primarily induced an increase in WBC levels in female animals at doses ≥ 25 mg/kg/day, a significant increase in NEUT, and a marked decrease in LYMPH in both male and female animals at doses ≥ 50 mg/kg/day. These changes exhibited statistical significance and showed a dose-related trend, suggesting that the administration of the test substance may influence WBC and its subcategories, NEUT and LYMPH.

The spleen and thymus are mainly composed of lymphoid tissue and important immune organs, making them common toxic target organs for drugs [20,21]. When damaged, usually a decrease in volume and weight manifests. In this study, we found that the volume of the spleen and thymus in female animals with a dose of hu7691 ≥ 25 mg/kg/day decreased, resulting in a decrease in organ weight and coefficient. Microscopic observation also showed a decrease in lymphocytes, thus affecting the immune system.

The digestive tract is the entry site into the body for orally administered test articles. An irritant test article may lead to local acute lesions at this first site of contact to the body and the digestive tract has been identified as the organ most commonly affected in British patients admitted to the hospital with adverse drug reactions (Pirmohamed et al., 2004) [22]. In this study, the gross anatomy of 3/3 male animals in the Hu7691 100 mg/kg/day dose group showed bleeding points in the glandular part of the stomach, as well as dilation of the duodenum and jejunum; histopathological examination revealed gastric submucosal edema and jejunal erosion, suggesting that administration of the test substance may affect the digestive system [22,23].

Compared to the solvent control group, male animals in the Hu7691 100 mg/kg/day group exhibited reduced absolute liver and kidney weights and relative brain weight coefficients. Male animals at 100 mg/kg/day and female animals at 75 mg/kg/day also showed decreased absolute heart weights and relative brain weight coefficients. In female animals at doses ≥25 mg/kg/day, there was a reduction in absolute ovarian weights and relative brain weight coefficients, although without a clear dose–response relationship. These changes in these indicators cannot exclude a potential association with the administration of the test substance. Subsequent long-term experiments should include histopathological examinations of the liver, kidneys, heart, and ovaries.

In summary, male Sprague Dawley (SD) rats were administered doses of 12.5, 50, 100, and 150 mg/kg/day, while female rats received doses of 12.5, 25, 50, and 75 mg/kg/day once daily for 14 consecutive days. Animal fatalities were observed in the male group at the 150 mg/kg/day dose and in the female group at the 50 mg/kg/day dose. The potential target organs for toxicity appear to be the spleen, thymus, and gastrointestinal tract, with the possibility of the test substance affecting the liver, kidneys, heart, and ovaries not excluded. Since hu7691 is a novel AKT inhibitor, although it has the above toxic damage, as a highly effective anti-tumor drug, it is still a promising clinical drug candidate.

## 5. Conclusions

In conclusion, male Sprague Dawley (SD) rats were administered doses of 12.5, 50, 100, and 150 mg/kg/day, while female rats received doses of 12.5, 25, 50, and 75 mg/kg/day once daily for 14 consecutive days. Animal fatalities were observed in the male group at the 150 mg/kg/day dose and in the female group at the 50 mg/kg/day dose. The potential target organs for toxicity appear to be the spleen, thymus, and gastrointestinal tract, with the possibility of the test substance affecting the liver, kidneys, heart, and ovaries not excluded. The NOAEL was determined to be no greater than 12.5 mg/kg/day. Based on the observed gender-related toxicity differences in the study, it is recommended that the high-dose reference dose for male animals in long-term experiments should not be less than 100 mg/kg/day, while for female animals, it should be less than 50 mg/kg/day.

## Figures and Tables

**Table 1:** Effect of Hu7691 on clinical symptoms after repeated administration for 14 days.

Dosage (mg/kg/Day)	Sex	Clinical Observations (a/b)					
		Bristles/Matte Hair	Red around the Nose	Perianal Filth	Hunched	Emaciated	Dead
0	♂	0/3	0/3	0/3	0/3	0/3	0/3
12.5	♂	0/3	0/3	0/3	0/3	0/3	0/3
50	♂	0/3	0/3	0/3	0/3	0/3	0/3
100	♂	3/3	2/3	0/3	3/3	0/3	0/3
150	♂	3/3	1/3	0/3	3/3	3/3	2/3
0	♀	0/3	0/3	0/3	0/3	0/3	0/3
12.5	♀	0/3	0/3	0/3	0/3	0/3	0/3
25	♀	0/3	1/3	0/3	1/3	1/3	0/3
50	♀	1/3	2/3	0/3	2/3	2/3	1/3
75	♀	3/3	3/3	1/3	3/3	3/3	1/3

Notes: a/b: the proportion animals of the symptoms in the total.

**Table 2:** List of hematology examination (values are mean ± SD for 3 rats/sex/ group).

Dosage (mg/kg/Day)	Sex		WBC (10^9^/L)	%NEUT (%)	%LYMPH (%)	%MONO (%)	%EOS (%)	RBC (10^12^/L)
0	♂		10.93 ± 1.82	7.3 ± 1.2	90.8 ± 1.3	1.4 ± 0.3	0.5 ± 0.2	7.12 ± 0.44
12.5	♂		6.91 ± 1.16	7.4 ± 2.6	90.4 ± 3.3	1.7 ± 1.0	0.5 ± 0.2	6.84 ± 0.42
50	♂		8.73 ± 1.70	11.0 ± 2.6 **	85.5 ± 3.7 *	3.0 ± 1.1	0.6 ± 0.3	7.54 ± 0.12
100	♂		11.83 ± 5.38	68.9 ± 6.1 ***	25.3 ± 5.2 ***	5.0 ± 2.9	0.7 ± 0.4	6.51 ± 1.33
0	♀		3.44 ± 2.23	7.6 ± 4.2	89.6 ± 5.1	1.7 ± 0.9	1.0 ± 0.1	7.22 ± 0.21
12.5	♀		3.78 ± 1.33	12.3 ± 8.8	85.2 ± 9.6	1.5 ± 0.2	1.0 ± 0.7	7.29 ± 0.53
25	♀		9.92 ± 2.65 *	23.2 ± 19.0	73.9 ± 20.1	1.9 ± 1.2	1.0 ± 0.3	6.67 ± 0.16 *
50	♀		10.01 ± 0.91 *	49.5 ± 1.6 **	46.3 ± 1.8 **	3.3 ± 0.0	0.9 ± 0.3	6.18 ± 0.66
75	♀		7.17 ± 3.15 *	53.7 ± 25.2 ***	42.9 ± 23.8 ***	2.0 ± 0.4	1.5 ± 1.1	5.68 ± 1.79
**Dosage (mg/kg/Day)**	**Sex**	**Hb** **(g/dL)**	**HCT** **(%)**	**MCV** **(fL)**	**MCH** **(Pg)**	**MCHC** **(g/dL)**	**PLT** **(10^9^/L)**	**RETIC** **(10^9^/L)**
0	♂	14.5 ± 0.3	42.5 ± 0.6	59.8 ± 2.7	20.4 ± 0.9	34.1 ± 0.2	1083 ± 31	432.9 ± 25.1
12.5	♂	14.1 ± 0.6	40.7 ± 1.3	59.6 ± 2.1	20.6 ± 0.5	34.6 ± 0.4 *	1036 ± 67	392.2 ± 53.3
50	♂	15.2 ± 0.3	43.7 ± 1.2	58.0 ± 0.7	20.2 ± 0.1	34.9 ± 0.3 **	981 ± 100 *	370.3 ± 47.3
100	♂	13.0 ± 2.7	36.0 ± 6.6	55.5 ± 1.2	19.9 ± 0.1	35.9 ± 1.0 *	1300 ± 304	376.5 ± 258.5
0	♀	13.9 ± 0.7	39.4 ± 1.5	54.6 ± 0.7	19.2 ± 0.4	35.1 ± 0.4	975 ± 65	275.0 ± 77.3
12.5	♀	14.7 ± 0.7	41.0 ± 1.6	56.4 ± 2.1	20.1 ± 0.8	35.8 ± 0.4	925 ± 51	250.2 ± 31.2
25	♀	13.7 ± 0.5 *	38.2 ± 1.4	57.3 ± 0.8 *	20.5 ± 0.2 *	35.7 ± 0.2 *	1085 ± 354	468.0 ± 97.7
50	♀	12.1 ± 1.7	34.7 ± 3.2	56.1 ± 0.8	19.6 ± 0.6	34.8 ± 1.7 **	1733 ± 296 *	444.7 ± 186.7
75	♀	11.1 ± 3.7	31.7 ± 10.5	55.6 ± 1.0	19.5 ± 0.3	35.1 ± 0.1 *	1566 ± 274	426.6 ± 287.0

Note: The significance levels observed are as follows: * *p* < 0.05; ** *p* < 0.01; *** *p* < 0.001 in comparison to control group values.

**Table 3:** List of serum chemistry examination (values are mean ± SD for 3 rats/sex/ group).

Dosage (mg/kg/Day)	Sex		TP (g/L)	Alb (g/L)	ALT (U/L)	AST (U/L)	TBIL (umol/L)	ALP (mmol/L)	BUN (umol/L)
0	♂		56.6 ± 1.7	41.7 ± 0.6	45.0 ± 5.9	147.9 ± 10.3	0.8 ± 0.3	230 ± 9	5.3 ± 1.0
12.5	♂		56.3 ± 1.7	41.9 ± 2.4	40.2 ± 5.9	106.4 ± 15.2 *	0.8 ± 0.5	249 ± 57	5.1 ± 1.6
50	♂		56.9 ± 0.1	39.9 ± 1.3	43.6 ± 10.2	137.3 ± 17.8	0.8 ± 0.4	162 ± 37 *	4.0 ± 0.5
100	♂		52.2 ± 1.6 *	29.5 ± 3.3 **	23.4 ± 8.9	99.4 ± 32.1	0.2 ± 0.3	106 ± 22 ***	10.3 ± 1.3 **
0	♀		60.1 ± 2.3	46.7 ± 2.2	32.9 ± 4.6	110.8 ± 10.8	1.4 ± 0.4	103 ± 21	6.7 ± 0.6
12.5	♀		62.2 ± 2.1	47.9 ± 2.8	33.9 ± 6.7	114.6 ± 16.0	1.7 ± 0.5	100 ± 3	6.8 ± 0.8
25	♀		61.2 ± 6.5	46.6 ± 5.7	34.7 ± 9.7	100.0 ± 11.2	1.1 ± 0.7	69 ± 34	9.2 ± 1.6
50	♀		46.3 ± 0.0 *	26.3 ± 0.6 **	37.0 ± 5.5	86.3 ± 11.2	0.8 ± 0.3	50 ± 2 *	10.4 ± 2.1
75	♀		56.3 ± 6.6	35.7 ± 9.1	38.7 ± 26.0	117.7 ± 22.7	1.4 ± 0.4	79 ± 21	12.1 ± 6.1
**Dosage (mg/kg/Day)**	**Sex**	**Cr** **(g/L)**	**Glu** **(g/L)**	**K** **(mmol/L)**	**NA** **(mmol/L)**	**CL** **(umol/L)**	**TG** **(mmol/L)**	**TC** **(mmol/L)**	**CK** **(umol/L)**
0	♂	26 ± 1	5.50 ± 0.71	4.90 ± 0.13	142 ± 1	100.5 ± 1.6	0.51 ± 0.20	1.25 ± 1.05	1156 ± 71
12.5	♂	26 ± 2	6.23 ± 0.40	4.64 ± 0.26	144 ± 1	102.4 ± 0.9	0.55 ± 0.13	0.55 ± 0.81	818 ± 232
50	♂	25 ± 4	5.97 ± 0.10	4.59 ± 0.09 *	143 ± 1	100.5 ± 0.2	1.36 ± 0.51	2.09 ± 0.32	1183 ± 122
100	♂	29 ± 2	4.81 ± 0.59	4.74 ± 0.23	143 ± 2	103.2 ± 1.1	1.22 ± 0.54	2.83 ± 0.59	702 ± 371
0	♀	38 ± 6	5.64 ± 0.26	4.15 ± 0.60	142 ± 2	99.6 ± 2.3	0.40 ± 0.12	1.38 ± 0.23	699 ± 42
12.5	♀	39 ± 2	6.32 ± 0.60	4.10 ± 0.07	143 ± 0	101.0 ± 1.1	0.38 ± 0.04	2.25 ± 0.29 *	806 ± 178
25	♀	40 ± 5	5.87 ± 0.59	3.88 ± 0.12	142 ± 2	98.9 ± 3.7	0.46 ± 0.08	1.96 ± 0.63	532 ± 158
50	♀	34 ± 4	4.90 ± 0.67	4.92 ± 0.05	142 ± 1	103.2 ± 1.6	2.55 ± 1.68	2.26 ± 0.24 *	688 ± 15
75	♀	36 ± 4	5.84 ± 1.07	4.76 ± 0.49	144 ± 1	102.0 ± 0.9	0.73 ± 0.08	2.52 ± 0.01 **	535 ± 313

Note: Compared with the control group, * *p* < 0.05, ** *p* < 0.01, *** *p* < 0.001.

**Table 4:** List of blood Coagulation Detection Index examination results (values are mean ± SD for 3 rats/sex/ group).

Dosage (mg/kg/Day)	Sex	APTT(s)	PT(s)
0	♂	8.0 ± 1.5	7.8 ± 0.3
12.5	♂	10.5 ± 0.4	7.8 ± 0.1
50	♂	9.0 ± 2.2	7.7 ± 0.1
100	♂	8.9 ± 0.5	7.7 ± 0.0
0	♀	9.8 ± 0.7	8.6 ± 0.6
12.5	♀	10.6 ± 3.1	9.1 ± 1.0
25	♀	9.4 ± 1.8	8.5 ± 0.2
50	♀	10.7 ± 10.1	8.1 ± 7.8
75	♀	11.4 ± 0.1	8.0 ± 0.4

Note: The significance levels observed are *p* > 0.05 in comparison to control group values.

**Table 5:** List of absolute organ weight (values are mean ± SD for 3 rats/sex/group).

Dosage (mg/kg/Day)	Sex		SPLEEN	LIVER	KIDNEY	ADRENALS	THYMUS
0	♂		0.7249 ± 0.0476	9.8091 ± 0.9568	2.5069 ± 0.0579	0.0428 ± 0.0100	0.7038 ± 0.1141
12.5	♂		0.6560 ± 0.0804	9.6923 ± 1.2075	2.4692 ± 0.1912	0.0395 ± 0.0030	0.5977 ± 0.1305
50	♂		0.5780 ± 0.0685 *	9.9034 ± 0.7158	2.3161 ± 0.1122	0.0403 ± 0.0056	0.3358 ± 0.1020 *
100	♂		0.2030 ± 0.0202 ***	6.6913 ± 0.4170 **	1.5625 ± 0.0597 ***	0.0300 ± 0.0052	0.0761 ± 0.0257 ***
0	♀		0.6360 ± 0.0877	7.7315 ± 0.6535	1.9792 ± 0.2029	0.0631 ± 0.0111	0.5214 ± 0.1420
12.5	♀		0.4255 ± 0.0225 *	6.8255 ± 0.1239	1.7739 ± 0.0321	0.0557 ± 0.0036	0.4555 ± 0.0892
25	♀		0.3973 ± 0.1707	6.3344 ± 0.7277	1.5645 ± 0.1521 *	0.0542 ± 0.0107	0.2082 ± 0.0900 *
50	♀		0.3414 ± 0.0619 *	6.6504 ± 0.3591	1.3779 ± 0.1180 *	0.0488 ± 0.0005	0.1233 ± 0.1055 *
75	♀		0.2458 ± 0.0670 *	6.4443 ± 0.0599	1.4232 ± 0.3043	0.0353 ± 0.0006 *	0.0970 ± 0.0851 *
**Dosage (mg/kg/Day)**	**Sex**	**HEART**	**BRAIN**	**TESTICLE**	**EPIDIDIMS**	**OVARIES**	**WOMB**
0	♂	1.2545 ± 0.0688	1.8932 ± 0.1410	2.6549 ± 0.0202	0.6507 ± 0.0917		
12.5	♂	1.2089 ± 0.0494	1.8676 ± 0.0664	2.8478 ± 0.1281	0.6631 ± 0.0919		
50	♂	1.1532 ± 0.0469	1.9459 ± 0.0988	2.8200 ± 0.4947	0.6440 ± 0.0882		
100	♂	0.8343 ± 0.0399 ***	1.9045 ± 0.0321	3.0220 ± 0.3458	0.6166 ± 0.0506		
0	♀	1.0607 ± 0.0609	1.9342 ± 0.0258			0.1396 ± 0.0049	0.3685 ± 0.0685
12.5	♀	0.9025 ± 0.0885	1.8307 ± 0.0090 **			0.1147 ± 0.0185	0.3968 ± 0.0501
25	♀	0.8658 ± 0.1433	1.7825 ± 0.1327			0.1126 ± 0.0099 *	0.3586 ± 0.1373
50	♀	0.8210 ± 0.1025 *	1.8012 ± 0.0477 *			0.0872 ± 0.0306	0.2941 ± 0.1203
75	♀	0.8776 ± 0.0246 *	1.9177 ± 0.0360			0.0864 ± 0.0081 **	0.2268 ± 0.0156

The significance levels observed are * *p* < 0.05, ** *p* <0.01 and *** *p* < 0.001 in comparison to control group values. *t*-test.

**Table 6:** List of relative organ weight/body weight (values are mean ± SD for 3 rats/sex/group).

Dosage (mg/kg/Day)	Sex		SPLEEN	LIVER	KIDNEY	ADRENALS	THYMUS
0	♂		0.2109 ± 0.0115	2.8544 ± 0.2808	0.7295 ± 0.0186	0.0125 ± 0.0030	0.2047 ± 0.0320
12.5	♂		0.1968 ± 0.0135	2.9350 ± 0.5525	0.7418 ± 0.0045	0.0119 ± 0.0006	0.1798 ± 0.0393
50	♂		0.1773 ± 0.0230 *	3.0327 ± 0.1533	0.7097 ± 0.0303	0.0124 ± 0.0020	0.1034 ± 0.0334 *
100	♂		0.0882 ± 0.0107 ***	2.9074 ± 0.2773	0.6774 ± 0.0030 **	0.0130 ± 0.0026	0.0328 ± 0.0097 ***
0	♀		0.2417 ± 0.0379	2.9308 ± 0.1816	0.7522 ± 0.0913	0.0240 ± 0.0043	0.1994 ± 0.0620
12.5	♀		0.1684 ± 0.0155 *	2.6983 ± 0.1569	0.7006 ± 0.0150	0.0220 ± 0.0023	0.1802 ± 0.0366
25	♀		0.1857 ± 0.0878	2.9489 ± 0.6734	0.7286 ± 0.1627	0.0248 ± 0.0037	0.0983 ± 0.0507
50	♀		0.1701 ± 0.0105	3.3385 ± 0.2218	0.6904 ± 0.0240	0.0246 ± 0.0032	0.0589 ± 0.0457
75	♀		0.1268 ± 0.0143 *	3.3985 ± 0.5195	0.7381 ± 0.0389	0.0186 ± 0.0027	0.0475 ± 0.0366
**Dosage (mg/kg/Day)**	**Sex**	**HEART**	**BRAIN**	**TESTICLE**	**EPIDIDIMS**	**OVARIES**	**WOMB**
0	♂	0.3651 ± 0.0219	0.2047 ± 0.0320	0.7725 ± 0.0052	0.1896 ± 0.0286		
12.5	♂	0.3645 ± 0.0302	0.1798 ± 0.0393	0.8575 ± 0.0476	0.1999 ± 0.0315		
50	♂	0.3536 ± 0.0197	0.1034 ± 0.0334 *	0.8643 ± 0.1515	0.1975 ± 0.0284		
100	♂	0.3616 ± 0.0054	0.0328 ± 0.0097 ***	1.3080 ± 0.1040 ***	0.2676 ± 0.0247 *		
0	♀	0.4027 ± 0.0274	0.1994 ± 0.0620			0.0530 ± 0.0040	0.1405 ± 0.0316
12.5	♀	0.3563 ± 0.0329	0.1802 ± 0.0366			0.0452 ± 0.0059	0.1573 ± 0.0263
25	♀	0.4017 ± 0.0932	0.0983 ± 0.0507			0.0518 ± 0.0047	0.1664 ± 0.0667
50	♀	0.4166 ± 0.1013	0.0589 ± 0.0457			0.0430 ± 0.0101	0.1445 ± 0.0428
75	♀	0.4642 ± 0.0880	0.0475 ± 0.0366			0.0452 ± 0.0031	0.1190 ± 0.0112

The significance levels observed are * *p* < 0.05, ** *p* <0.01 and *** *p* < 0.001 in comparison to control group values. *t*-test.

**Table 7:** List of relative organ weight/brain weight (values are mean ± SD for 3 rats/sex/group).

Dosage (mg/kg/Day)	Sex	SPLEEN	LIVER	KIDNEY	ADRENALS	THYMUS
0	♂	38.3281 ± 1.4699	518.1590 ± 34.5187	132.8023 ± 7.9521	2.2943 ± 0.7151	37.0121 ± 3.5065
12.5	♂	35.2493 ± 5.5822	518.7932 ± 58.5405	132.5686 ± 15.1094	2.1220 ± 0.2353	32.0979 ± 7.5437
50	♂	29.7780 ± 4.2084 *	508.6051 ± 11.9040	119.1164 ± 5.4313	2.0837 ± 0.3797	17.4630 ± 6.0305 **
100	♂	10.6696 ± 1.2342 ***	351.6505 ± 28.0513 **	82.0323 ± 2.3705 ***	1.5767 ± 0.2993	3.9878 ± 1.2975 ***
0	♀	32.8470 ± 4.1430	399.7410 ± 33.6162	102.3844 ± 11.2781	3.2684 ± 0.6109	26.9221 ± 7.0784
12.5	♀	23.2407 ± 1.1581*	372.8430 ± 6.4049	96.9035 ± 2.0308	3.0424 ± 0.1934	24.8936 ± 4.9750
25	♀	22.0046 ± 8.6217	355.1793 ± 29.4804	87.8005 ± 6.6001	3.0611 ± 0.7300	11.5635 ± 4.7535 *
50	♀	18.9152 ± 2.9385*	369.0860 ± 10.1691	76.4363 ± 4.5296	2.7078 ± 0.0991	6.7703 ± 5.6781 *
75	♀	12.8502 ± 3.7331*	336.1378 ± 9.4321	74.3752 ± 17.2626	1.8414 ± 0.0641	5.0982 ± 4.5316 *
**Dosage (mg/kg/day)**	**Sex**	**HEART**	**TESTICLE**	**EPIDIDIMS**	**OVARIES**	**WOMB**
0	♂	66.6880 ± 8.6860	140.7071 ± 9.5896	34.6570 ± 6.6415		
12.5	♂	64.8128 ± 4.2740	152.7603 ± 12.1614	35.5677 ± 5.4317		
50	♂	59.3902 ± 4.4840	144.9807 ± 25.1509	33.1594 ± 4.9988		
100	♂	43.7945 ± 1.4480 *	158.6207 ± 17.0399	32.4007 ± 3.0500		
0	♀	54.8691 ± 3.7782			7.2153 ± 0.2069	19.0245 ± 3.2897
12.5	♀	49.2874 ± 4.6447			6.2701 ± 1.0382	21.6684 ± 2.6783
25	♀	48.4052 ± 5.3772			6.3180 ± 0.3267 *	19.8641 ± 6.5851
50	♀	45.6720 ± 6.9008			4.8176 ± 1.5724	16.2426 ± 6.2479
75	♀	45.7604 ± 0.4243 *			4.5077 ± 0.5086 **	11.8367 ± 1.0334

The significance levels observed are * *p* < 0.05, ** *p* <0.01 and *** *p* < 0.001 in comparison to control group values. *t*-test.

## Data Availability

The data that support the findings of this study are available on request from the corresponding authors.

## References

[1] Anderson W., Barker A.D., Bell C., Bhan M. (2010). International network of cancer ge-nome projects. Nature.

[2] Nitulescu G.M., Margina D., Juzenas P., Peng Q., Olaru O.T., Saloustros E.-M., Fenga C., Spandidos D.A., Libra M., Tsatsakis A.M. (2016). Akt inhibitors in cancer treatment: The long journey from drug discovery to clinical use. Int. J. Oncol..

[3] He Y., Sun M.M., Zhang G.G., Yang J., Chen K.S., Xu W.W., Li B. (2021). Targeting PI3K/Akt signal transduction for cancer therapy. Signal Transduct. Target. Ther..

[4] Blake J.F., Xu R., Bencsik J.R., Xiao D., Kallan N.C., Schlachter S., Mitchell I.S., Spencer K.L., Banka A.L., Wallace E.M. (2012). Dis-covery and Preclinical Pharmacology of a Selective ATPCompetitive Akt Inhibitor (GDC-0068) for the Treatment of Human Tumors. J. Med. Chem..

[5] Uko N.E., Güner O.F., Matesic D.F., Bowen J.P. (2020). Akt Pathway Inhibitors. Curr. Top. Med. Chem..

[6] Jeong S.-H., Kim H.-B., Kim M.-C., Lee J.-M., Lee J.H., Kim J.-H., Kim J.-W., Park W.-Y., Kim J.B., Kim H. (2018). Hippo-mediated suppression of IRS2/AKT signaling prevents hepatic steatosis and liver cancer. J. Clin. Investig..

[7] Lin Q., Wang Y., Chen D., Sheng X., Liu J., Xiong H. (2017). Cisplatin regulates cell autophagy in endometrial cancer cells via the PI3K/AKT/mTOR signalling pathway. Oncol. Lett..

[8] Lee J.B., Jung M., Beom S.H., Kim G.M., Kim H.R., Choi H.J., Sohn J.H., Ahn J.B., Rha S.Y., Chung H.C. (2021). Phase 2 study of TAS-117, an allosteric akt inhibitor in advanced solid tumors harboring phosphatidylinositol 3-kinase/v-akt murine thymoma viral oncogene homolog gene mutations. Investig. New Drugs.

[9] Roudsari N.M., Lashgari N.-A., Momtaz S., Abaft S., Safaiepour P., Narimisa K., Jackson G., Bishayee A., Rezaei N., Bishayee A. (2021). Inhibitors of the PI3K/Akt/mTOR Pathway in Prostate Cancer Chemoprevention and Intervention. Pharmaceutics.

[10] Ghoneum A., Said N. (2019). PI3K-AKT-mTOR and NF_B Pathways in Ovarian Cancer: Implications for Targeted Therapeutics. Cancers.

[11] McKenna M., McGarrigle S., Pidgeon G.P. (2018). The next generation of PI3K-Akt-mTOR pathway inhibitors in breast cancer cohorts. BBA—Rev. Cancer.

[12] Zhang J., Yu X.H., Yan Y.G., Wang C., Wang W.J. (2015). PI3K/Akt signaling in osteosarcoma. Clin. Chim. Acta.

[13] Park J.-Y., Lin P.-Y., Weiss R.H. (2007). Targeting the PI3K-Akt pathway in kidney cancer. Expert Rev. Anticancer Ther..

[14] Simpson D.R., Mell L.K., Cohen E.E.W. (2015). Targeting the PI3K/AKT/mTOR pathway in squamous cell carcinomaof the head and neck. Oral Oncol..

[15] Yang J., Cron P., Thompson V., Good V.M., Hess D., Hemmings B.A., Barford D. (2002). Molecular Mechanism for the Regulation of Protein Kinase B/Akt by Hydrophobic Motif Phosphorylation. Mol. Cell.

[16] Luci I., Rathinaswamy M.K., Truebestein L., Hamelin D.J., Burke J.E. (2018). Conformational sampling of membranes by Akt controls its activation and inactiva-tion. Proc. Natl. Acad. Sci. USA.

[17] Bing S., Xiang S., Xia Z., Wang Y., Guan Z., Che J., Xu A., Dong X., Cao J., Yang B. (2023). AKT inhibitor Hu7691 in-duces differentiation of neuroblastoma cells. Acta Pharm. Sin. B.

[18] Delwatta S.L., Gunatilake M., Baumans V., Seneviratne M.D., Dissanayaka M.L.B., Batagoda S.S., Udagedara A.H., Walpola P.B. (2018). Reference values for selected hematological, biochemical and physiological parameters of Sprague-Dawley rats at the Animal House, Faculty of Medicine, University of Colombo, Sri Lanka. Animal Model Exp. Med..

[19] Che J., Dai X., Gao J., Sheng H., Zhan W., Lu Y., Li D., Gao Z., Chen B., Luo P. (2021). Discovery of N-((3S,4S)-4-(3,4-Difluorophenyl)piperidin-3-yl)-2-fluoro-4-(1-methyl-1H-pyrazol-5-yl)benzamide (Hu7691), a Potent and Selective Akt Inhibitor That Enables Decrease of Cutane-ous Toxicity. J. Med. Chem..

[20] Bahceci İ., Tumkaya L., Mercantepe T., Aslan N., Duran Ö.F., Soztanaci U.S., Yazıcı Z.A. (2023). Inhibition of methotrexate induced toxicity in the adult rat spleen by adalimumab. Drug Chem. Toxicol..

[21] Willard-Mack C.L., Elmore S.A., Hall W.C., Harleman J., Kuper C.F., Losco P., Rehg J.E., Rühl-Fehlert C., Ward J.M., Weinstock D. (2019). Nonproliferative and Proliferative Lesions of the Rat and Mouse Hematolymphoid System. J. Toxicol. Pathol..

[22] Pirmohamed M., James S., Meakin S., Green C., Scott A.K., Walley T.J., Park B.K., Breckenridge A.M. (2004). Adverse drug reactions as cause of admission to hospital: Prospective analysis of 18 820 patients. BMJ.

[23] Nolte T., Brander-Weber P., Dangler C., Deschl U., Elwell M.R., Greaves P., Hailey R., Leach M.W., Pandiri A.R., Rogers A. (2016). Nonproliferative and Proliferative Lesions of the Gastrointestinal Tract, Pancreas and Salivary Glands of the Rat and Mouse. J. Toxicol. Pathol..

